# Machine learning approach for early onset dementia neurobiomarker using EEG network topology features

**DOI:** 10.3389/fnhum.2023.1155194

**Published:** 2023-06-16

**Authors:** Tomasz M. Rutkowski, Masato S. Abe, Tomasz Komendzinski, Hikaru Sugimoto, Stanislaw Narebski, Mihoko Otake-Matsuura

**Affiliations:** ^1^RIKEN Center for Advanced Intelligence Project, Tokyo, Japan; ^2^The University of Tokyo, Tokyo, Japan; ^3^Nicolaus Copernicus University, Toruń, Poland; ^4^Doshisha University, Kyoto, Japan

**Keywords:** EEG, dementia, biomarker, mild cognitive impairment, machine learning, artificial intelligence, prevention, network neuroscience

## Abstract

**Introduction:**

Modern neurotechnology research employing state-of-the-art machine learning algorithms within the so-called “AI for social good” domain contributes to improving the well-being of individuals with a disability. Using digital health technologies, home-based self-diagnostics, or cognitive decline managing approaches with neuro-biomarker feedback may be helpful for older adults to remain independent and improve their wellbeing. We report research results on early-onset dementia neuro-biomarkers to scrutinize cognitive-behavioral intervention management and digital non-pharmacological therapies.

**Methods:**

We present an empirical task in the EEG-based passive brain-computer interface application framework to assess working memory decline for forecasting a mild cognitive impairment. The EEG responses are analyzed in a framework of a network neuroscience technique applied to EEG time series for evaluation and to confirm the initial hypothesis of possible ML application modeling mild cognitive impairment prediction.

**Results:**

We report findings from a pilot study group in Poland for a cognitive decline prediction. We utilize two emotional working memory tasks by analyzing EEG responses to facial emotions reproduced in short videos. A reminiscent interior image oddball task is also employed to validate the proposed methodology further.

**Discussion:**

The proposed three experimental tasks in the current pilot study showcase the critical utilization of artificial intelligence for early-onset dementia prognosis in older adults.

## 1. Introduction

Late-age cognitive decline, beginning with mild cognitive impairment (MCI) and often leading to dementia, caused mainly by Alzheimer's syndrome (AS) (Herrup, 2021) or vascular dementia spectrum of neurodegenerative diseases, is an actual healthcare emergency exemplified by evolved mental impairment in older adults with a span of psychological or behavioral symptoms (Livingston et al., 2020). Until now, there is no viable non-invasive biomarker helping to predict a possible early onset of MCI or even dementia, nor a pharmacological intervention stopping the disease progress, and only a postmortem autopsy is the conclusive determination (Herrup, 2021). Still, modern late-age dementia decline diagnostics comprises paper and pencil examinations such as a Montreal Cognitive Assessment (MoCA) (Fujiwara et al., 2010). MoCA scores 25 and below (MoCA ≤ 25) define MCI onset. There are several trials to develop an objective examination brain monitoring techniques focusing on non-invasive EEG (Rutkowski et al., 2020b, 2021a,b, 2022a,b) concurrently with behavioral evaluations (Rutkowski et al., 2020a). Such timely research for a neurodegenerative decline, particularly MCI, prediction is an essential scientific topic but still in the emerging research stages (WHO, 2019; Myszczynska et al., 2020; Shi et al., 2022).

Aging-societies-related dementia case increases represent a substantial and rapidly rising load on the healthcare ecosystem (WHO, 2019; Livingston et al., 2020). Contemporary societies expect the feasible attention of AI research to focus on possible diagnostics and non-pharmacological-therapeutic (NPT) approaches (Zucchella et al., 2018) in order to aid the wellbeing of aging communities. Our present report illustrates an application of a wearable headset Unicorn EEG by g.tec medical engineering GmbH, Austria. We focus our research on retail wearable headsets to capture EEG shortly in home-based environments, considering substantial environmental electromagnetic noise and no clinical experimental experience of the target older adult users. The wearable EEG headbands have already been proven satisfactory in academic research (Barachant et al., 2019; Rutkowski et al., 2022b).

Contemporary neurotechnology applications such as brain-computer interfaces (BCI) and efficient machine learning (ML) algorithms improve the daily lives of individuals with limited mobility or communication skills (Guger et al., 2015). An extension of the neurotechnology applications to a field of neuro-biomarkers of age-related cognitive decline and early onset dementia opens new opportunities to monitor cognitive-behavioral interventions (Otake-Matsuura et al., 2021) and digital non-pharmacological therapies (NPT; Sikkes et al., 2020). We discuss a unique experimental task in an EEG-based passive BCI application framework to evaluate working memory, which estimates MCI prediction. We report findings from two older adult volunteer groups in Poland of the proposed cognitive decline prediction task by analyzing EEG responses to short facial emotion-displaying videos. The EEG responses are analyzed in a framework of network neuroscience using an ordinal partition network (OPN) approach (Varley et al., 2021; Varley and Sporns, 2022).

A positive result of increased lifespan globally is associated with chronic late-age-related illnesses such as cognitive decline (Livingston et al., 2020). Fortunately, a possible application of digital health technologies, home-based self-management, and the development of novel screening tests for assessing cognitive dysfunction in older adults might help minimize the negative impact of healthcare systems (WHO, 2019). An introduction of home-based self-assessment and self-management approaches for older adults with cognitive dysfunction is of critical importance. Our research project aims to develop simple EEG wearable-based neurotechnology for easy self-evaluation and possible cognitive or lifestyle intervention monitoring.

Behavioral studies have shown that recognizing facial expressions may be impaired in patients with AS. There is already literature reporting studies on electrophysiological indicators of face recognition disorders in patients with AS (Güntekin et al., 2019), which provides the basis for future research to elucidate the behavioral and neural basis of facial emotional processing in AS (Fide et al., 2019). These non-linear methods, especially the calculation of permutation entropy values, offer opportunities for discrimination leading to the identification of AS. MCI and AS result in less variability and complexity in brain dynamics. Moreover, these advanced analysis methods can already apply to existing EEG data for future research. Thus, reducing EEG complexity could be considered a marker for detecting AS. The development of such a classification will give a chance to use this classification as a clinical tool (Şeker et al., 2021). The studies cited above may be the basis for research on emotional processing in patients with various types of dementia. The goal here will be to discover electrophysiological indices helpful in clinical practice as correlates of emerging behavioral problems.

Lately, there has been a flurry of research inspiration in modeling the brain as a network, with nodes indicating brain regions or single neurons and edges resembling structural or statistical dependencies (Bullmore and Sporns, 2009). Morabito et al. (2015) suggested a complex network technique integrated with time dynamics to perform a time-space investigation to illustrate the progression of AS in longitudinal studies. However, a recently rising discipline of network neuroscience focusing on the so-called network analysis of time series (Varley et al., 2021; Varley and Sporns, 2022) has permitted researchers to leverage the substantial force of graph theory and network science to analyze neural manifolds for temporal brain microstate number elucidation. Varley and Sporns (2022) in a recent review on network analysis of brainwave time series such as ECoG, LFP, or EEG, have shown that a complementary branch of network neuroscience, which focuses on an analysis of temporal data structures instead of functional or structural connectivity networks, could be a novel tool in computational neuroscience. Such a novel approach to network analysis of time series allows for collapsing at a given instant signal into a single state vector with edges corresponding to movement via state space. The edges could be directed or undirected, weighted, or not.

We propose three unique experimental tasks allowing for EEG recordings that consider the evaluation of working/short-term during facial emotion assessment learning, evaluation, and reminiscent interior oddball tasks. We next apply network analysis of EEG time series to elucidate differences between healthy aging cognition and MCI in older adult participants. We acknowledge the limitation of the current study concerning a restricted number of older adult participants in the reported pilot study. The presented encouraging results in the leave-one-out-subject cross-validation setting shall possibly be soon reproduced with a larger older adult participants cohort. The motivation and details of the proposed experimental procedure are explained in subsequent sections. We also develop novel EEG processing and machine learning classification procedures, which we describe in methods focusing sections. The results presentation and future application discussion conclude the paper.

### 1.1. Working/short-term memory in healthy and MCI-concerned older adults

Among late and middle-aged adults, self-reported short-term memory problems often signify intermediate and long-term risk factors of vascular and all-cause dementia (Möllers et al., 2022). Working-memory impairments often occur in MCI patients and a further decline in dementia-diagnosed individuals, indicating that working memory evaluation is a good candidate for assessment as part of neuropsychological diagnostics of age-related cognitive decline possibly leading to dementia (Kessels et al., 2011).

As mentioned earlier, the reports indicate impaired working memory in age-related cognitive decline with a gradient between MCI and dementia (Gagnon and Belleville, 2011) are feasible candidates for behavioral and neurotechnological experimental tasks leading to digital neuro-biomarker development. We propose including working memory learning of a new skill in the proposed emotion assessment learning and evaluation task as introduced in Sections 2.1.1 and 2.1.2.

### 1.2. Reminiscent image interventions for the older adults

Reminiscence is a non-pharmacological intervention technique employed to manage the behavioral and psychological symptoms of dementia (Khait et al., 2021). Park et al. (2019) reported depression symptom reduction and increased quality of life in older adults after applying reminiscence intervention. A reminiscence is an act of recalling one's past experiences and affairs. Personal memories define one's identity by connecting past events with the future (Buzsáki et al., 2022). Reminiscence intervention or stimulation is an interaction that involves communicating past life events utilizing tangible audiovisual aids such as photos, music, or videos (Thomas and Sezgin, 2021). In the current study, we employ a previously developed by our research group (Rutkowski et al., 2021a, 2022b) reminiscent interior photography/images oddball task to assess the working memory of older adult subjects and subsequent development of EEG neuro-biomarker as explained in Section 2.1.3.

### 1.3. Facial emotion recognition and visuospatial learning in healthy and MCI-concerned older adults

Facial emotion recognition and emotional intelligence improve with age (Gutchess, 2019). Blessing et al. (2010) reported that even in subjects with severely impaired explicit memory, implicit learning of affective responses (e.g., valence and arousal ratings) is still possible in patients with dementia. The above report suggests that a facial emotion assessment task is feasible for evaluating short-term memory learning skills of affective responses in MCI-declining and dementia-diagnosed older adult individuals since emotion recognition is a stable trait even in dementia, but the short-term memory declines (Gutchess, 2019). Therefore we propose to include facial emotion evaluation assessment learning and testing tasks to utilize implicit learning and short-term memory characteristics in healthy cognitive aging vs. MCI participants (see Sections 2.1.1 and 2.1.2 for details).

Alescio-Lautier et al. (2007) reported that visual recognition memory and specific attentional mechanisms are impaired in early dementia of AS type. The authors concluded that a combination of attentional and visuospatial evaluation should be a viable direction for discovering predictive neuro-biomarkers distinguishing MCI individuals from those converting to dementia. A report by Seo et al. (2018) further suggested that including visuospatial reproduction and working memory would also facilitate early detection of MCI. The two above studies inspired our research team to include a visuospatial task in the experimental task, a skill-learning task to evaluate facial emotions with an emoji-grid first proposed by Toet et al. (2018), as explained in Sections 2.1.1 and 2.1.2.

## 2. Methods

EEG experimental data collection with the older adult volunteering participants was accomplished at the Nicolaus Copernicus University in Torun, Poland, in the summer of 2022. The Institute of Psychology UNC Ethical Committee for Experiments with Human Subjects has endorsed the investigation. The experimental procedure and information collection adhered to The Declaration of Helsinki, regulating ethical principles for research concerning human subjects, including the investigation of identifiable human material and data. In the study, 27 older adult participants took part with a mean age of 70.76 ± 5.34 years old (for detailed age distribution, see Supplementary Figure 1), single male and remaining female participants. In the initial group of 27 participants, 18 older adults were MCI and the remaining nine of healthy cognitive aging (see Supplementary Figures 2–4 for detailed MoCA score distribution in each experimental task). All participants volunteered for the study and signed informed consent forms.

### 2.1. Stimulus presentation

Taking into account previous research findings related to working memory, facial emotion recognition, visuospatial learning, and reminiscence, as summarized in Sections 1.1–1.3 we create three simple cognitive tasks for the older adult subjects.

#### 2.1.1. Emotion evaluation learning task

**Figure 1:**
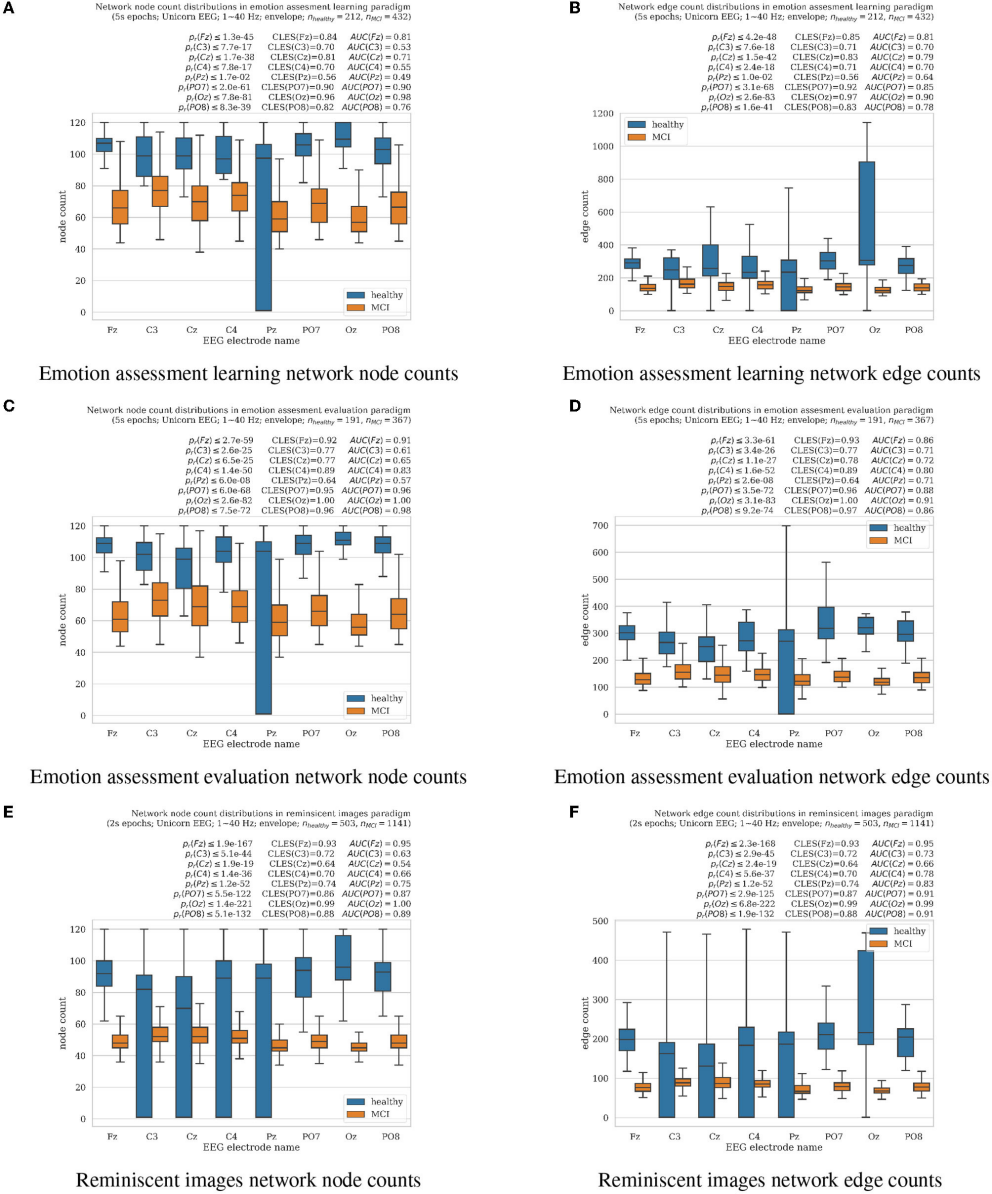
Boxplots with marked median, quartile ranges, and whiskers extending to show the rest of the distributions (all non-normal) of the network signal analysis resulting in node and edge counts for MCI vs. healthy aging cognition subjects for all EEG electrodes analyzed separately (Unicorn EEG headset with eight *Fz*, *C*3, *Cz*, *C*4, *Pz*, *PO*7, *Oz*, and *PO*8 scalp locations). **(A, B)** represent results from emotion assessment learning, **(C, D)** from emotion assessment evaluation, and **(E, F)** reminiscent interior images oddball tasks, respectively. Wilcoxon rank-sum test for significantly differing distributions resulting *p*_*r*_-values together with the common language effect size (CLES) (McGraw and Wong, 1992) and area under the ROC curve (AUC) (Hanley and McNeil, 1982) scores are summarized for each electrode over each panel, respectively.

**Figure 2:**
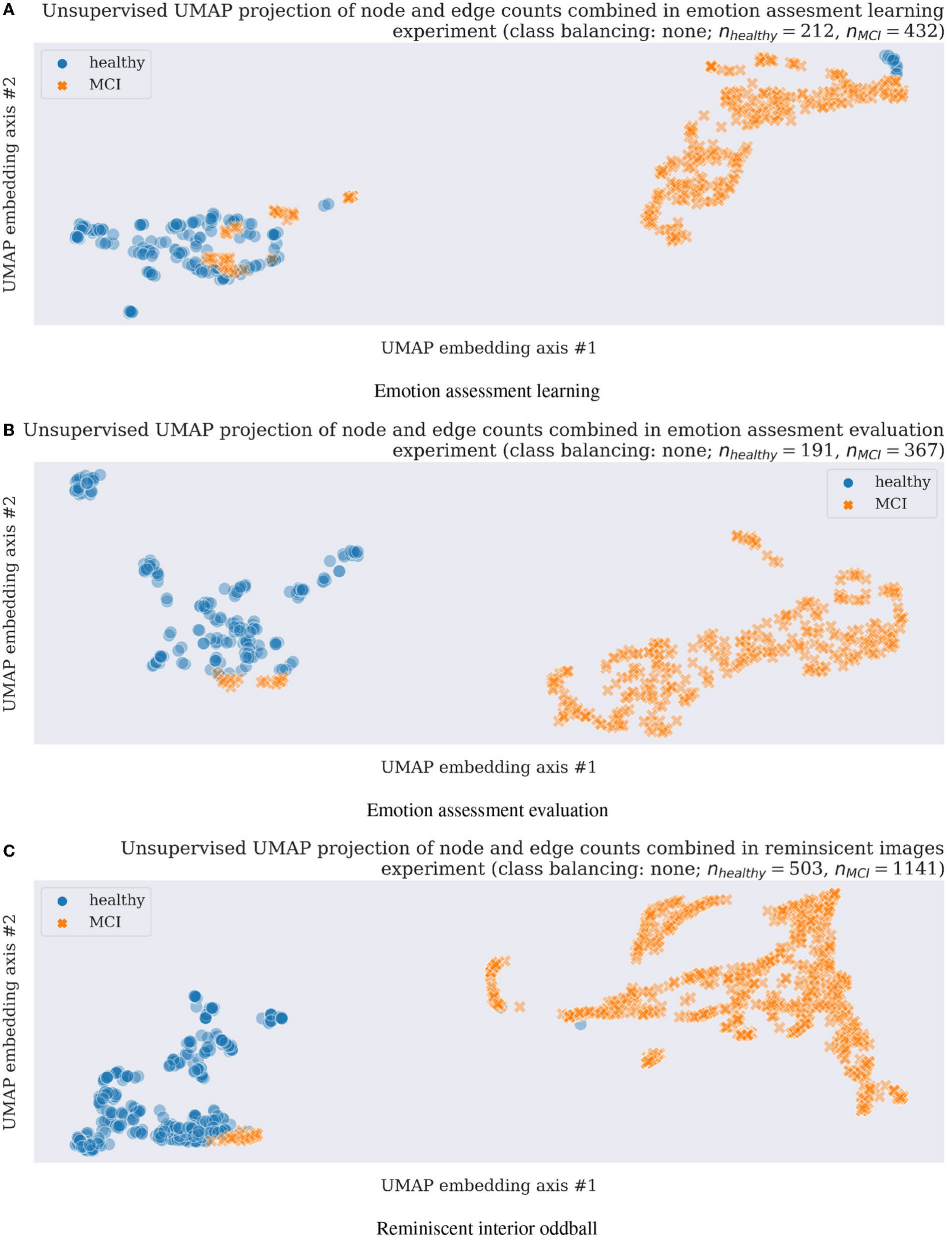
Unsupervised clustering (a machine learning training without class labels) scatter plots using UMAP in three experimental tasks and original data without any data augmentation, thus with unbalanced classes as shown with *n*_*healthy*_ vs. *n*_*MCI*_ feature numbers above each scatterplot. **(A)** Emotion assessment learning. **(B)** Emotion assessment evaluation. **(C)** Reminiscent interior oddball.

The stimulus presentation protocol is the same as in the previously published by our research group employing Japanese participants (Rutkowski et al., 2020a, 2021b). This time, each Polish older adult sitting in front of a display presenting short facial emotion videos from a Mind Reading database (Baron-Cohen, 2004) is instructed to also observe a two-dimensional grid of valence and arousal (Toet et al., 2018; Rutkowski et al., 2020a, 2021b) and later, after the end of each video, to input similar score on the same design grid on a touchpad. This task involves facial emotion assessment evaluation learning and visuospatial memory elements. In the reported project, we record continuous EEG with triggers marking all stimulus presentation and participant response stages from 27 older adults. We present 24 videos (5 s each on average), from the Mind Reading database (Baron-Cohen, 2004), for each participant covering valence and arousal for positive and negative scores (six in each quadrant of a two-dimensional grid; Toet et al., 2018). An experimental session consists of 24 emotional video clip presentations resulting in 24 responses contributed by each subject (212 from healthy and 432 from MCI participants in total after rejecting responses with missing markers due to stimulus system or network errors, as explained with *n*_*healthy*_ and *n*_*MCI*_ variables in the top panels of Figures 1, 2).

#### 2.1.2. Emotion evaluation assessment task

In the subsequent task, we instruct the participants to test how they learned to evaluate the short facial emotion display videos. This time they are instructed to input their valence and arousal evaluation on the same touchpad grid without a preceding suggestion prompt (Toet et al., 2018; Rutkowski et al., 2020a, 2021b). In this case, we also record continuous EEG with triggers marking all stimulus presentation and participant response stages from 24 older adults in the reported project. Similarly, as we have done in an emotion assessment evaluation learning task, we also present 24 videos (5 s each on average), from the Mind Reading database (Baron-Cohen, 2004), for each participant covering valence and arousal for positive and negative scores (six in each quadrant of a two-dimensional grid; Toet et al., 2018). An experimental session consists of 24 emotional video clip presentations resulting in 24 responses contributed by each subject (191 from healthy and 367 from MCI participants in total after rejecting responses with missing markers due to stimulus system or network errors, as explained with *n*_*healthy*_ and *n*_*MCI*_ variables in the middle panels of Figures 1, 2).

#### 2.1.3. Reminiscent interior images oddball task

In order to evaluate working memory in older adults, for dementia neuro-biomarker development purposes, we modify a standard oddball task to include childhood reminiscent interior images (Rutkowski et al., 2022b). Each short experimental trial presents eight types of modern and participants' childhood-time interior photographs. As in the classical oddball task, each stimulus from the series of eight became a target once, and participants are instructed to remember it before each trial. Here too, we record continuous EEG with triggers marking all experimental stages from 23 older adults in the reported project. Each participant session contains a presentation of eight oddball sessions containing eight randomly ordered interior images (four reminiscent and four modern rooms). An experimental session consists of 8 oddball sessions consisting of a single interior image (a target) presentation followed by eight presentations with a randomly positioned target photograph, thus, resulting in 72 responses contributed by each subject (503 from healthy and 1, 141 from MCI participants in total after rejecting responses with missing markers due to stimulus system or network errors, as explained with *n*_*healthy*_ and *n*_*MCI*_ variables in the bottom panels of Figures 1, 2).

### 2.2. EEG capture

We collect EEG data in the current study using a Unicorn EEG headset by g.tec Medical Engineering, Austria. The Unicorn EEG headset has already been proven a reliable experimental device, compared to other available wearables, in our previously published studies (Rutkowski et al., 2022c). For the initial investigation, we use eight EEG channels uniformly covering the human scalp at the standard locations of (*Fz*, *C*3, *Cz*, *C*4, *Pz*, *PO*7, *Cz*, and *PO*8). The eight EEG streams initially digitized with a sampling frequency of 250 Hz are bandpass filtered in the first preprocessing step to remove signal baseline shifts and high-frequency noise within a frequency band of 1–40 Hz. We next segment (“epoch”) the EEG signals using video and image stimulus onset recorded triggers in emotion assessment and reminiscent interior tasks for five- and two-second epochs, respectively. We implement the filtering and segmentation procedures using the MNE package ver. 1.3.0 (Gramfort et al., 2014) in Python ver. 3.10.9. In the next step, to remove eye-blink and muscle movement-related artifacts in the collected EEGs, we employ a previously developed methodology by the members of the current research team (Rutkowski et al., 2008; Rutkowski and Mori, 2015). EEG channels are decomposed into intrinsic mode functions (IMF) using an empirical mode decomposition (EMD) method, and all the components that exceed the 100 μV threshold we reject before the final signal reconstruction from sub-threshold IMFs. We implement the above EEG cleaning procedures in PyEMD ver.1.4.0 (Laszuk, 2017). We next rectify the resulting filtered EEG traces to extract amplitude envelope traces using a Hilbert transform (SciPy ver. 1.10.0; Virtanen et al., 2020 implementation) and pass them to the network neuroscience application to time series, as explained in the next section.

### 2.3. Network science approach to EEG time-series

Permutation sequences in a time series are sensitive characteristics of the dynamic state of an observed system (Bandt and Pompe, 2002). They can be efficiently computed even for long time-series EEG. One crucial advantage of the permutation analysis approach is the possibility of mapping a continuous EEG recording to a little cluster of discrete permutations. Subsequently, it is possible to apply principled information-theoretic approaches, such as permutation entropy (Bandt and Pompe, 2002).

Varley et al. (2021) proposed an exploration of temporal dynamics of an observed system to derive ordinal partition network (OPN) representations of recorded neuropsychological data (EEG in our case). We also apply a similar procedure, and to avoid problems with multivariate OPNs, we analyze each EEG channel separately, and afterward, we combine the obtained network characteristics as input to final classifiers. Such a methodology allows for limitations of possible remaining EEG artifacts (eye-blinks, etc.) impact on the analysis, and each electrode cortical-region-related network features separate examination with limitations related to the spatial EEG resolution.

**Figure 3:**
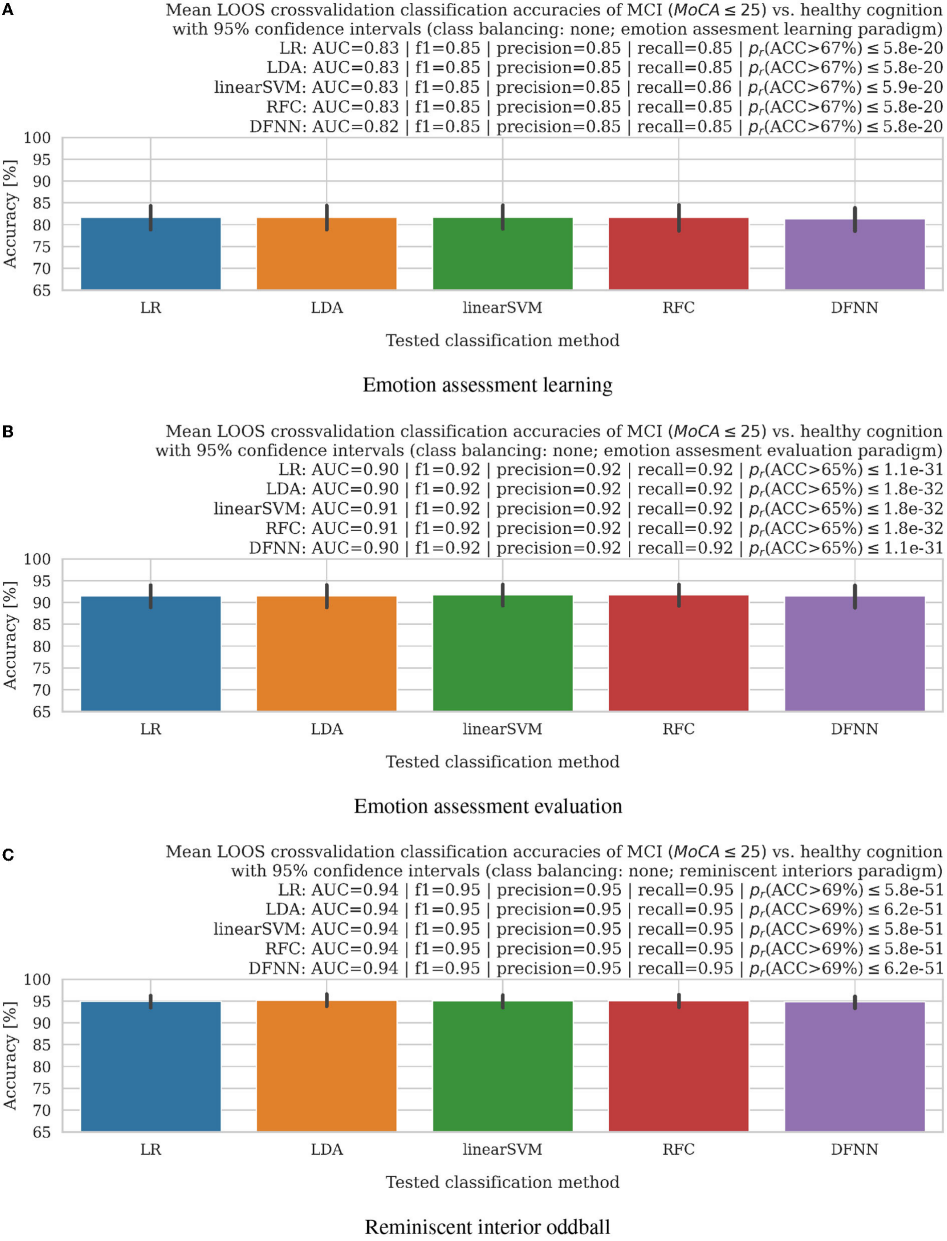
Bar plots with 95% confidence intervals of mean accuracies in leave-one-subject-out (LOOS) cross-validation setting of MCI vs. healthy aging cognition subjects using logistic regression (LR), a linear discriminant analysis (LDA), linear support vector machine (linearSVM), random forest (RFC), and deep fully-connected neural network (DFNN) classifiers. AUC, f1-scores, precision, recall, and Wilcoxon rank-sum test for significance *p*_*r*_-values (all non-normal distributions) of the accuracy distributions above training set chance levels (denoted in parentheses for all experimental settings separately), which we listed above the bar plots, further supported good results of the proposed methodology. **(A)** Emotion assessment learning. **(B)** Emotion assessment evaluation. **(C)** Reminiscent interior oddball.

To create an OPN model characterizing EEG time series from a single channel *c*, we assemble a vector **X**_*c*_ = *x*_*c*, 1_, *x*_*c*, 2_, …, *x*_*c, n*_, for limited time points *t* = 1, …, *n* in a single experimental trial to be analyzed next. The so obtained data vector is next embedded in *d*−dimensional space, utilizing τ time-lag. We select τ using the first zero crossing of the autocorrelation for each EEG channel time series representing an experimental trial. We limit a search for optimal τ in a range of 0–80 ms for computational and research reproducibility reasons. For the same reasons, we also specify the embedding dimension to *d* = 5 (similarly as in examples published by Varley et al., 2021), for results visualization purposes as shown in Figures 1, 2, as well as in the final supervised learning classification results reported in Figure 3. The resulting temporally ordered *d*-length vectors υ_*c, i*_ = [*x*_*c, i*_, *x*_*c, i*+τ_, …, *x*_*c, i*+(*d*−1)τ_] are next mapped to the permutation π to sort, in increasing order, their coefficients. The positions of the coefficients are next replaced in ordering π, and the resulting new vector *n*_*c, i*_ = [π(*x*_*c, i*_), π(*x*_*c, i*+τ_), …, π(*x*_*c, i*+(*d*−1)τ_)] represents a permutation of the numbers 1, 2, …, *d*. These permutations we consider as nodes in a directed network graph. The nodes we connect with directed edges and create from consecutive points. The so-created transition network results with fewer nodes than the initially analyzed EEG channel time series since there might be many *i* for which the delay vectors υ_*i*_ result in the same permutation π. The final OPN receives weights to each node representing a count of time points *i* representing the same permutation. At our project's current stage, we characterize each EEG channel *c* with a number of nodes and edges modeling each experimental trial (the so-called EEG epoch). The open-source package OPyN (Varley et al., 2021) has been used in our project to compute network neuroscience applications to EEG time series.

### 2.4. Unsupervised machine learning for network features visualization and dimensionality reduction

Unsupervised machine learning methods allowing for a datasets dimensionality reduction and visualization such as a uniform manifold approximation and projection (UMAP; McInnes et al., 2018) assume the available features (in the current project case, those are network node and edge counts from analyzed EEG electrode time-series separately as explained in Section 2.3; thus the final inputs to unsupervised model contain eight-node and edge counts obtained from each EEG responses and concatenated together, resulting in 16-dimensional feature vectors) are uniformly distributed across a topological manifold. The manifold could be approximated from these finite network features and projected to a lower-dimensional space. In the presented study, we apply the UMAP technique first in unsupervised learning mode to visualize EEG-derived network neuroscience features (Varley et al., 2021; Varley and Sporns, 2022) separability with results presented in Section 3.2. Next, a supervised learning mode of UMAP (McInnes et al., 2018) is applied for the network neuroscience features' dimensionality reduction in a leave-one-out-subject cross-validation classification to prove further the proposed methodology validation for future healthy cognitive aging vs. MCI early diagnostics with results discussed in Section 3.2.

### 2.5. Supervised machine learning models for leave-one-out-subject cross-validation of healthy aging vs. MCI EEG classification

In order to finally evaluate the usability of the proposed network neuroscience application to EEG time series for the early onset dementia (MCI with MoCA ≤ 25) elucidation, we employ several machine learning models in the leave-one-out-subject cross-validation (LOOS) setting. Similarly, as in the case of the unsupervised model discussed in Section 2.4 here, initial supervised machine learning input features are likewise composed of the network node and edge counts concatenated for all analyzed EEG electrode time-series separately as explained in Section 2.3 creating 16-dimensional integer-value-features, which are next reduced to eight dimensions using a supervised version of UMAP methodology explained in Section 2.4. The LOOS approach allows for keeping in each cross-validation step all data of a single subject and training a model using all the remaining subjects; thus, the procedure could be repeated for all the available subjects and final accuracies are concatenated and averaged, with a standard deviation calculation as discussed in Section 3.3.

In the reported study, we first applied UMAP supervised dimensionality reduction (McInnes et al., 2018) and next the following machine learning models (see Table 1 for details), available in the scikit-learn ver. 1.2.0 (Pedregosa et al., 2011) for classification: logistic regression (LR), linear discriminant analysis (LDA), linear kernel support vector machine (linearSVM), random forest classifier (RFC), deep fully connected neural network (DFNN).

**Table 1:** Supervised machine learning models employed in the study for binary classification of healthy aging cognition vs. early onset dementia (MCI) (Pedregosa et al., 2011).

**Machine learning (ML) model name**	**Parameters**
LR—logistic regression	Liblinear solver
LDA—linear discriminant analysis	Solver using least-squares
linearSVM—linear support vector machine	Squared hinge loss; *l*2−penalty
RFC—random forest classifier	Maximum depth of 15
DFNN—deep fully connected neural network	Six layers of 8, 256, 245, 128, 32, 16 ReLU units (RU);
	two softmax units; early stopping; ADAM optimizer

## 3. Results

The results of the present study could be summarized three-fold. Firstly, we have shown that the network neuroscience approach to EEG time series resulted in statistically significantly differing node and edge counts. Secondly, applying the unsupervised machine learning clustering UMAP technique visualized a clear separation of network neuroscience application to EEG time series features from healthy cognitive aging and MCI participants in three simple cognitive experimental tasks. Finally, the leave-one-out-subject cross-validation evaluation of supervised machine learning classifiers resulted in mean classification outcomes that were significantly above chance levels.

### 3.1. Network neuroscience node and edge distribution results

Results of EEG time-series analysis with the OPN technique described in Section 2.3 were summarized in the form of the network node and edge count distributions as shown in Figure 1. For all introduced in this paper, experimental cognitive tasks of facial emotion assessment learning (Section 2.1.1) and evaluation (Section 2.1.2), as well as reminiscent interior images (Section 2.1.3), healthy cognitive aging participants resulted in significantly higher results comparing to MCI cases as evaluated in non-parametric Wilcoxon rank-sum tests at *p*_*r*_≪0.01, except for *Pz* electrode for which *p*_*r*_ < 0.05 for nodes in emotion assessment tasks. All results did not follow normal distributions (normality tests failed with *p*_*n*_ < 0.05 for all); thus, non-parametric statistical significance outcomes we supported by reliable common-language-effect-size (CLES) (McGraw and Wong, 1992) and area under the ROC curve (AUC) (Hanley and McNeil, 1982) evaluations indicated above all panels in Figure 1. The *Pz* electrode location has been known for difficulties in clear EEG recording due to larger hair volumes, especially in female subjects, who were a majority in the reported experiments. Limiting the number of EEG electrodes shall also contribute to a more comfortable experimental setup for the older adult participants. The significantly higher node and edge network counts refer to higher consciousness levels as previously reported by Varley et al. (2021) in anesthetized vs. awake animals. In the current study, the MCI subject EEG analysis resulted in significantly lower node and edge numbers than the healthy cognition participants, as shown in Figure 1. In previous animal study (Varley et al., 2021), results reported in anesthesia-modulated consciousness also resulted in lower network node and edge counts of analyzed brainwaves in lower awareness. Therefore, we hypothesize that the MCI group's EEG in our study might suggest lower stages of participant awareness than the healthy aging cognition group. The results also illustrated a less predictable EEG signal structure in healthy cognitive aging participants or more flexible than in MCI cases.

### 3.2. Unsupervised UMAP clustering results

A subsequent application of unsupervised UMAP clustering technique resulted in the majority of cases separation as visualized in Figure 2 for all three experimental tasks described in Sections 2.1.1–2.1.3. The very encouraging results on a still limited participant group of 27 in emotion assessment learning (Section 2.1.1), 24 emotion assessment evaluation (Section 2.1.2), and 23 in the final reminiscent interior oddball (Section 2.1.3) supported the project hypothesis of network neuroscience methodology feasibility as a strong candidate for early onset dementia neuro-biomarker development. In order to address the problem of imbalanced datasets (almost double of MCI cases compared to healthy participants), we resembled results with data augmentation steps using majority under- and minority class over-sampling steps as implemented using a synthetic minority over-sampling technique (SMOTE; Chawla et al., 2002; Lema^ıtre et al., 2017). We summarized the still easily separable clustering results in Supplementary Figures 5, 6. Future research steps shall confirm the preliminary findings with a more extensive and preferably multicultural research group focusing on dementia level regression or multi-class or -cluster approaches.

### 3.3. Supervised leave-one-out-subject cross-validation classification of MCI vs. healthy aging cognition cases

The very encouraging results in the LOOS setting modeled a future real-world neuro-biomarker application, in which a machine learning model would be trained on a known, limited dataset and next applied to an unknown brainwave dataset for diagnostic purposes. The LOOS supervised machine learning accuracy results applied to four shallow- and one deep-learning models, as explained in Section 2.5, were summarized in Figure 3. Here again, to address the problem of imbalanced datasets (almost double of MCI cases compared to healthy participants), we resembled results with data augmentation steps using majority under- and minority class over-sampling steps as implemented using a synthetic minority over-sampling technique (SMOTE; Chawla et al., 2002; Lema^ıtre et al., 2017). The data augmentation-based class balancing results did not vary significantly, as summarized in Supplementary Figures 7, 8. For the case of the facial emotion assessment learning (Section 2.1.1), mean accuracies were safely above chance levels of 67% as imposed by training data imbalance and summarized in the top panel of Figure 3 with following results: *ACC*_*LR*_ = 81.63% (83.24% for class-balance over-sampling and 83.31% for under-sampling), *ACC*_*LDA*_ = 81.63% (83.24% for class-balance over-sampling and 83.08% for under-sampling), *ACC*_*linearSVM*_ = 81.69% (83.24% for class-balance over-sampling and 83.08% for under-sampling), *ACC*_*RFC*_ = 81.64% (83.24% for class-balance over-sampling and 83.08% for under-sampling), *ACC*_*DFNN*_ = 81.34% (83.24% for class-balance over-sampling and 83.08% for under-sampling); for all the cases median accuracies were at 100% level for LR, LDA, linearSVM, RFC, and DFNN (see Section 2.5 for details), respectively. Similarly, mean accuracy results in the facial emotion assessment evaluation task (see Section 2.1.2 for details) were safely above chance levels of 65% as imposed by training data imbalance and summarized in the middle panel of Figure 3 with following results: *ACC*_*LR*_ = 91.53% (92.56% for class-balance over-sampling and 92.82% for under-sampling), *ACC*_*LDA*_ = 91.57% (92.56% for class-balance over-sampling and 92.61% for under-sampling), *ACC*_*linearSVM*_ = 91.78% (92.56% for class-balance over-sampling and 92.61% for under-sampling), *ACC*_*RFC*_ = 91.78% (92.56% for class-balance over-sampling and 92.82% for under-sampling), *ACC*_*DFNN*_ = 91.53% (92.56% for class-balance over-sampling and 92.61% for under-sampling); for all the cases median accuracies were at 100% level for LR, LDA, linearSVM, RFC, and DFNN (see Section 2.5 for details), respectively. Finally, mean accuracy results in the reminiscent interior oddball task (see Section 2.1.3 for details) were safely above chance levels of 69% as imposed by training data imbalance and summarized in the bottom panel of Figure 3 with following results: *ACC*_*LR*_ = 94.94% (95.81% for class-balance over-sampling and 95.48% for under-sampling), *ACC*_*LDA*_ = 95.20% (93.74% for class-balance over-sampling and 95.48% for under-sampling), *ACC*_*linearSVM*_ = 95.02% (95.81% for class-balance over-sampling and 95.48% for under-sampling), *ACC*_*RFC*_ = 95.09% (95.72% for class-balance over-sampling and 95.48% for under-sampling), *ACC*_*DFNN*_ = 94.84% (95.53% for class-balance over-sampling and 95.48% for under-sampling); for all the cases median accuracies were at 100% level for LR, LDA, linearSVM, RFC, and DFNN (see Section 2.5 for details), respectively. The excellent supervised learning and LOOS cross-validated accuracy results with median accuracies reaching 100% levels for all the proposed cognitive tasks further supported a choice of network neuroscience approaches as the very reliable dementia neuro-biomarker prospects.

## 4. Discussion

Previous application of network neuroscience analysis to brainwave time series resulted in consciousness level association with network edge and node numbers (Varley et al., 2021) in anesthetized animals. Higher consciousness levels were associated with the larger node and edge numbers modeling brainwave time series, pointing to higher complexity and more brain microstates associated with those cognitive states (Varley et al., 2021). Consciousness is closely related to awareness (Ehret and Romand, 2022); thus, results presented in the current study with conscious (awake) subjects clearly show significantly lower network node and edge counts in MCI participants, indicating lower awareness levels compared to healthy cognitive aging older adults. In the presented study, more significant numbers of node and edge numbers in networks modeling EEG in healthy cognitive aging participants elucidated those brains characterized by more affluent microstates during cognitive tasks designed for the current study. Three different experimental tasks introduced in the study resulted in a larger number of modeling network nodes and edges for healthy cognitive aging vs. MCI older adults, further confirming a stable neuro-biomarker candidate, as summarized in Section 3.1.

The reproduced results in three cognitive tasks also elucidate potentially sound characteristics of the network neuroscience neuro-biomarker prospect. The statistical significance of network node and edge distribution differences was also confirmed with the unsupervised machine learning clustering UMAP approach as presented in Section 3.2.

The resulting unsupervised clusters formed easily separable quantities for most analyses used in the study. The final LOOS cross-validation experiment presented in Section 3.3 indicated a possible subsequent candidate for the following study with a more significant number of participants to infer all MoCA or other cognitive scores and not only binary healthy vs. MCI stages, as in the currently reported project.

The inherent study limitation has been a still low number of subjects (27, 24, and 23 in the three evaluated tasks) and unbalanced class membership (double the number of MCI compared to healthy cognitive aging). A single male participant also limited the study from the gender impact evaluation perspective. A near-future project with better gender-balanced subjects and possibly in cross-cultural settings shall be conducted to reproduce and validate the results. Another limitation of the current study, due to a low number of participants, has been a binary class membership (MCI vs. healthy cognitive aging). A subsequent study with more participants shall aim at continuous cognitive state estimation by predicting (regressing) exact MoCA or other cognitive state evaluations. A final limitation of the current study was a lack of relation of the predicted MCI stages based on only MoCA scores. The proposed neuro-biomarker shall be further validated with PET and cerebrospinal fluid (CSF) biomarkers for AS or structural MRI for vascular dementia evaluation.

## 5. Conclusions

This work discusses how network neuroscience methods' application to EEG time series can elucidate the separation between two distinct states of age-related healthy cognition and MCI. To assess each EEG channel's temporal dynamics, we assemble discrete state-transition graphs employing the ordinal partition networks approach, demonstrating how the brain evolves through state space in time. We discover that the healthy cognitive aging condition is characterized by a high degree of within each EEG channels interactions. Additionally, a less predictable EEG signal structure, or more flexible, is observed in healthy cognitive aging participants compared to MCI. Finally, unsupervised and supervised machine learning approaches allow us to separate and classify network neuroscience features for possible subsequent diagnostics of early onset dementia (MCI with MoCA ≤ 25) onset. The work is a step forward in developing a low-cost, home-based neuro-biomarker to monitor cognitive interventions and dementia care management.

## Data availability statement

The datasets presented in this article are not readily available because, the raw EEG data generated and analyzed for this study cannot be shared due to participant privacy protection purposes. Requests to access the datasets should be directed to tomasz.rutkowski@riken.jp.

## Ethics statement

The studies involving human participants were reviewed and approved by the Institute of Psychology UNC Ethical Committee for Experiments with Human Subjects. The patients/participants provided their written informed consent to participate in this study.

## Author contributions

TR conceived the concept of the working memory emotional learning and assessment, as well as reminiscent interior images oddball tasks, designed and programmed experimental stimulus presentation, EEG acquisition, analysis, and wrote the manuscript. TR and MA proposed network neuroscience to EEG time-series application for subsequent classification using unsupervised and supervised machine learning methods. TK, SN, and TR recruited and managed the subjects, as well as conducted the experiments. TR, MA, SN, TK, and HS interpreted the results. TR, MA, HS, SN, TK, and MO-M examined outcomes. All authors contributed to the article and approved the submitted version.
