# Supplementary material for: Machine learning approach for early onset dementia neurobiomarker using EEG network topology features
Source: Front Hum Neurosci. 2023 Jun 16;17:1155194. doi: 10.3389/fnhum.2023.1155194 (PMC10311997; doi:10.3389/fnhum.2023.1155194)
Supplement: Supplementary file 1 [file Data_Sheet_1.PDF]

frontiers

Supplementary Material

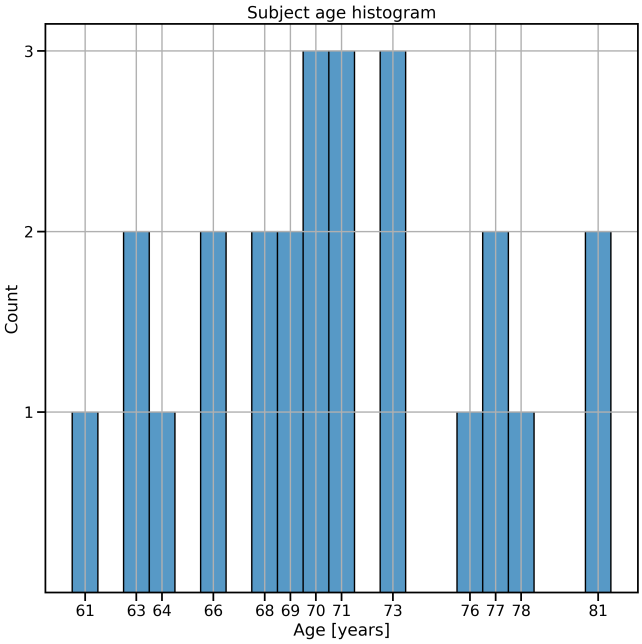

Figure S1. The elderly 27 participants' age histogram.

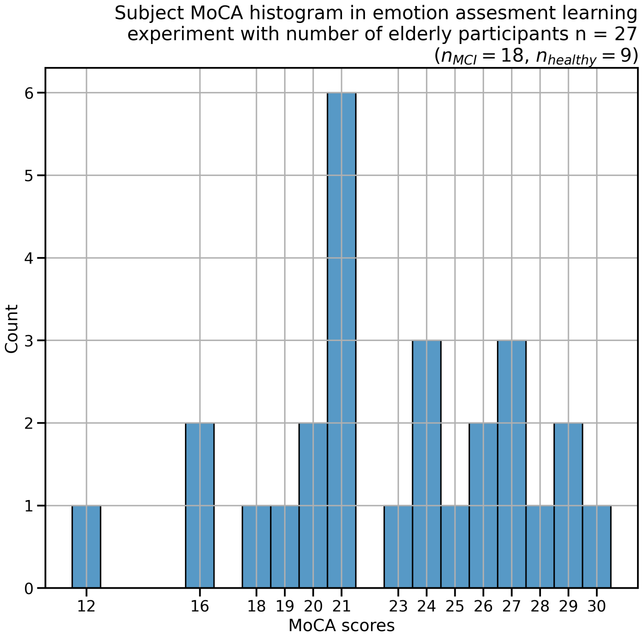

Figure S2. The elderly 27 participants' histogram of MoCA scores in the emotion assessment learning task.

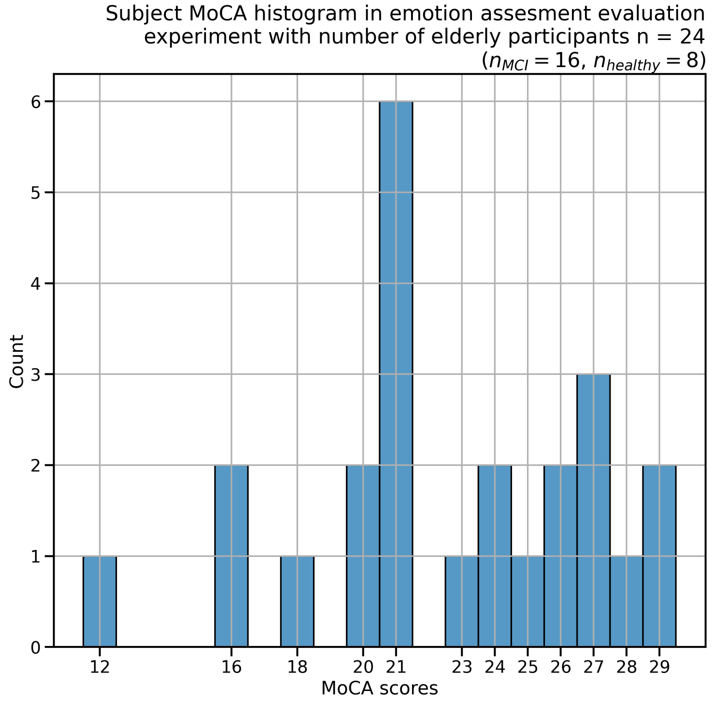

Figure S3. The elderly 24 participants' histogram of MoCA scores in the emotion assesment evaluation task.

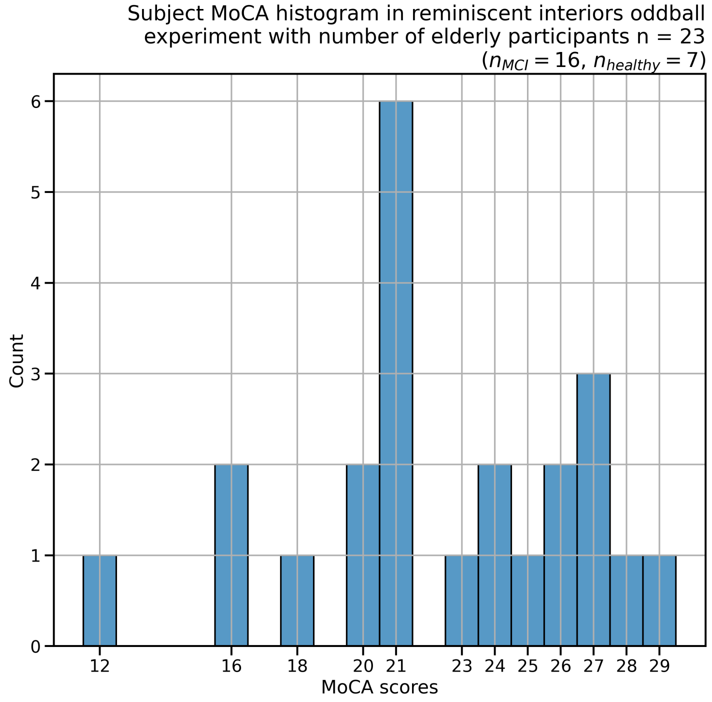

Figure S4. The elderly 23 participants' histogram of MoCA scores in the reminiscent interior photography oddball task.

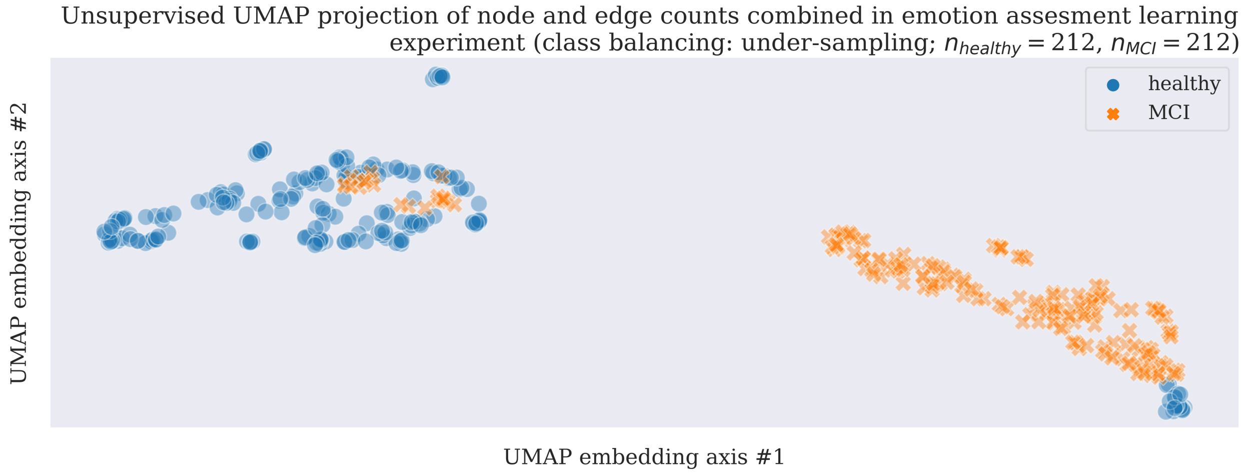

(a) Emotion assessment learning

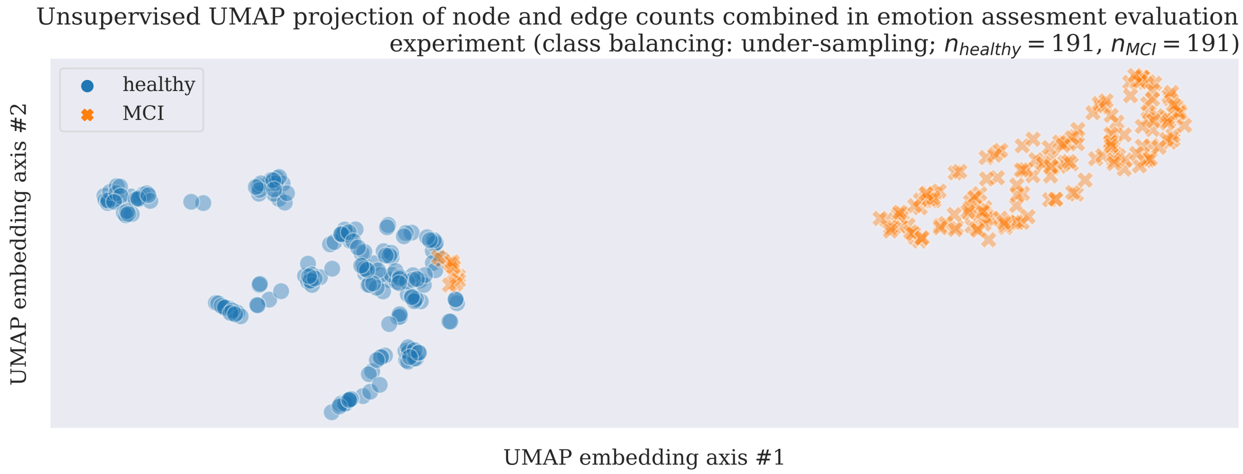

(b) Emotion assessment evaluation

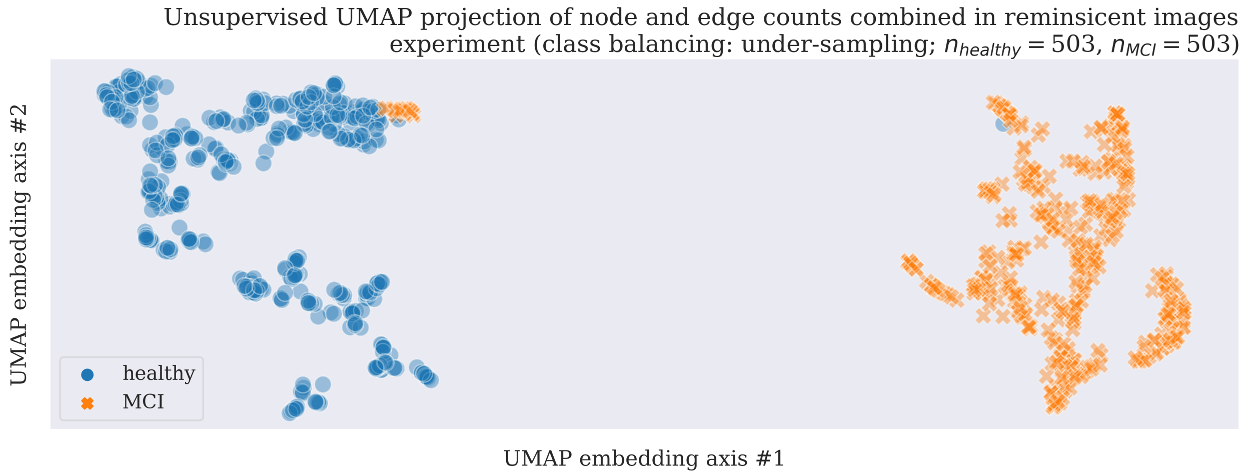

(c) Reminiscent interior oddball

Figure S5. Unsupervised clustering (a machine learning training without class labels) scatter plots using UMAP in three experimental tasks and balanced/under-sampled data augmentation using random under-sampling (Lemaître et al., 2017), thus creating balanced classes as shown with $n_{healthy}$ versus n_{MCI} feature numbers above each scatterplot, and a subsequent chance level of 50%. The under-sampling data augmentation creates clusters similar to the orignal datasets depiced in Figure 2.

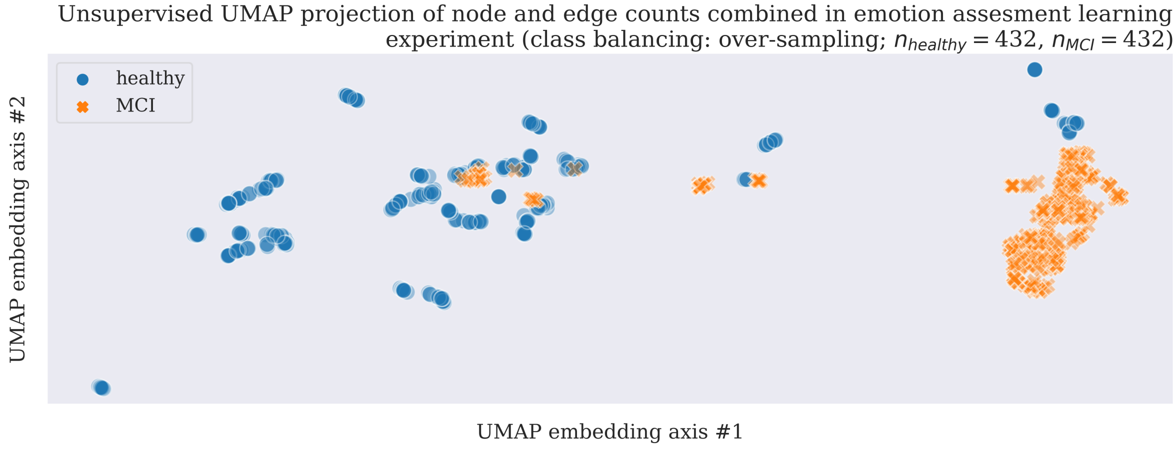

(a) Emotion assessment learning

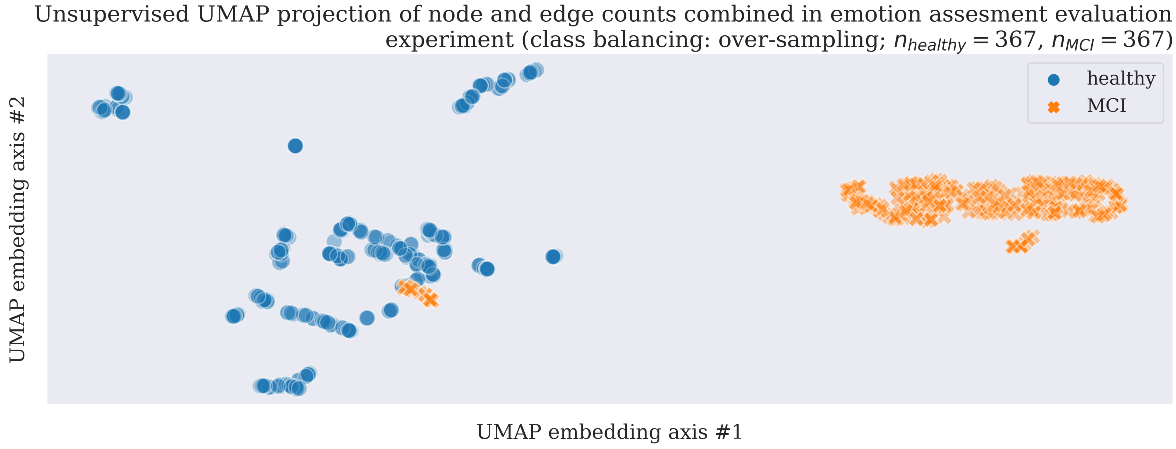

(b) Emotion assessment evaluation

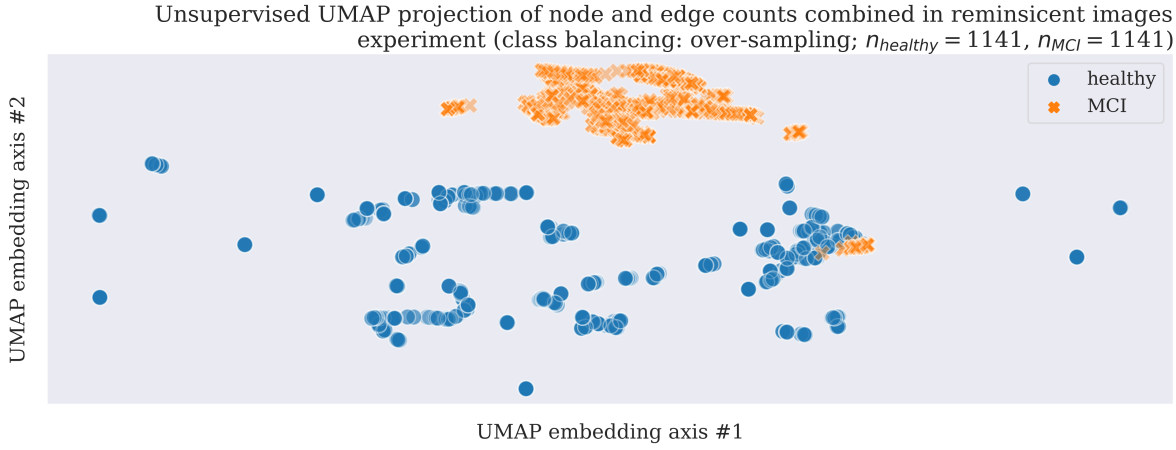

(c) Reminiscent interior oddball

Figure S6. Unsupervised clustering (a machine learning training without class labels) scatter plots using UMAP in three experimental tasks and balanced/over-sampled data augmentation using random under-sampling (Lemaître et al., 2017), thus creating balanced classes as shown with $n_{healthy}$ versus n_{MCI} feature numbers above each scatterplot, and a subsequent chance level of 50%. The over-sampling data augmentation (randomly multiplying samples in a minority class (Lemaître et al., 2017)) creates cluster distortions compared to the original datasets clusters depicted in Figure 2.

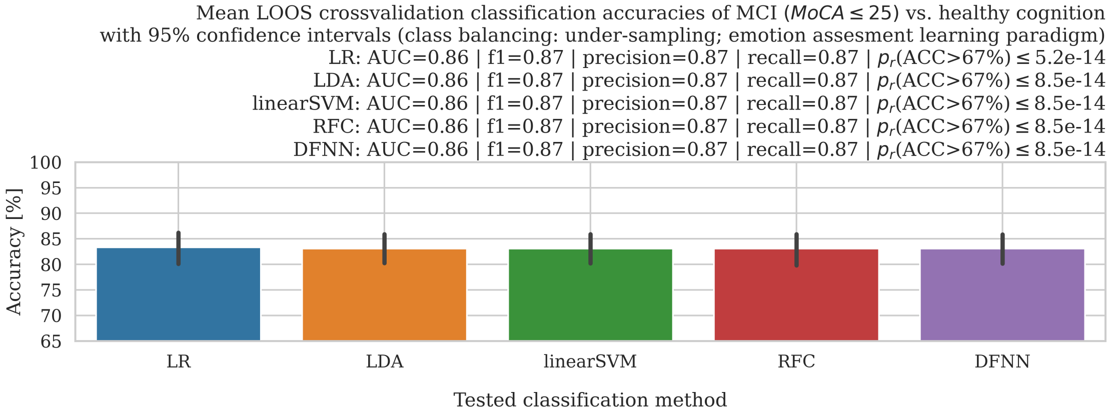

(a) Emotion assessment learning

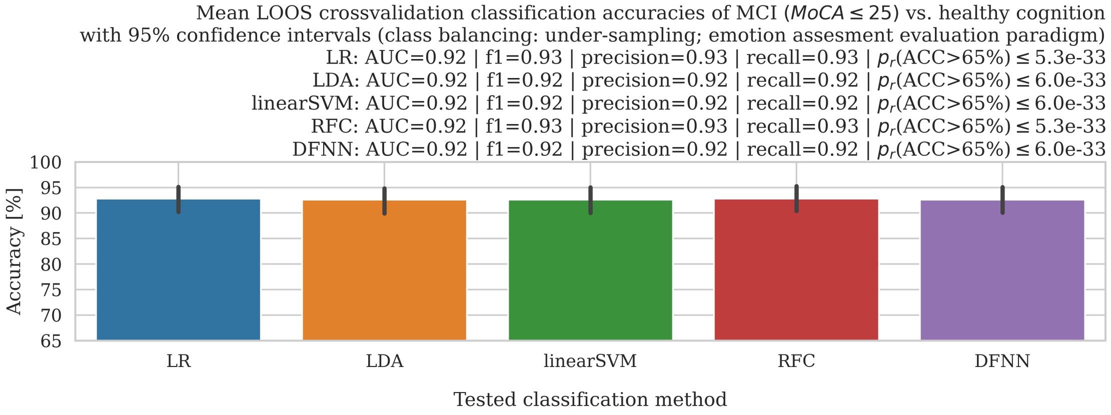

(b) Emotion assessment evaluation

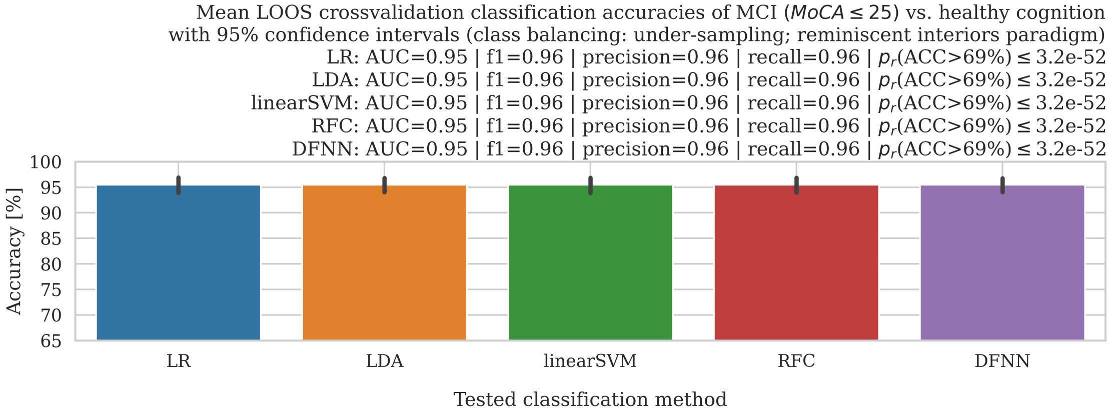

(c) Reminiscent interior oddball

Figure S7. Bar plots with 95% confidence intervals of mean accuracies in leave-one-subject-out (LOOS) cross-validation setting of MCI versus healthy aging cognition subjects using logistic regression (LR), a linear discriminant analysis (LDA), linear support vector machine (linearSVM), random forest (RFC), and deep fully-connected neural network (DFNN) classifiers. AUC, f1-scores, precision, recall, and Wilcoxon rank-sums test for significance p-values (all non-normal distributions) of the accuracy distributions above training set chance levels, which we listed above the bar plots, further supported good results of the proposed methodology. The under-sampling data augmentation (Lemaître et al., 2017) resulted in similar mean accuracies and remaining classification result metrics as in the original datasets depicted in Figure 3. Thus the under-sampling data augmentation did not significantly influence classification results.

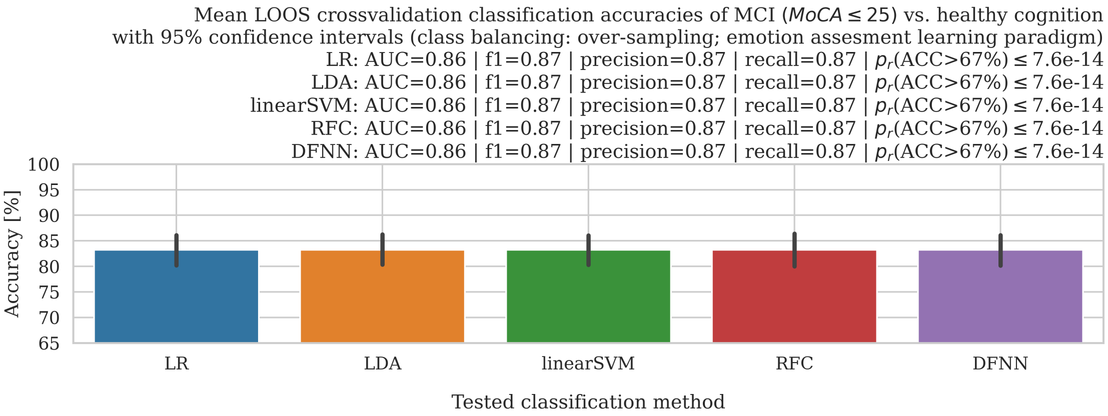

(a) Emotion assessment learning

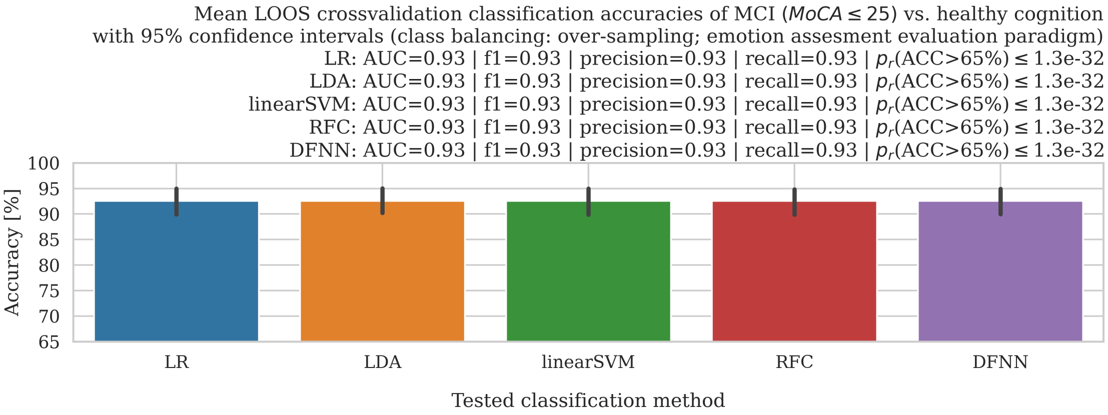

(b) Emotion assessment evaluation

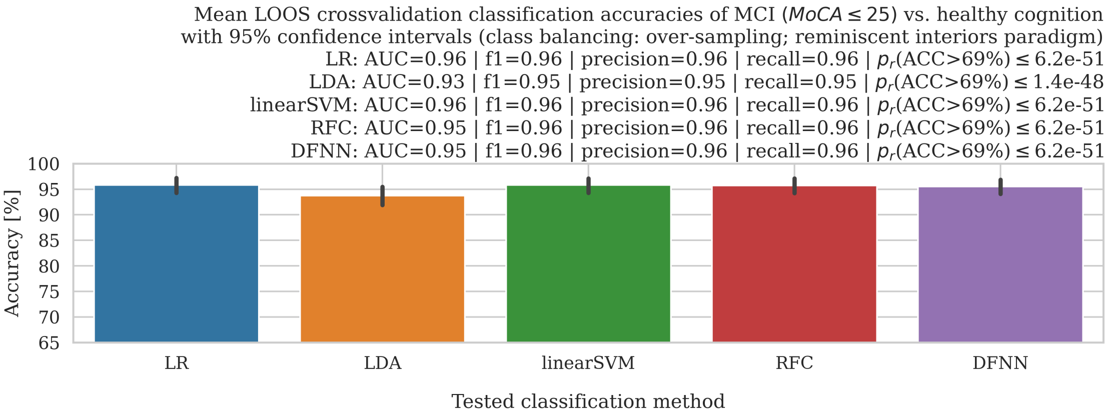

(c) Reminiscent interior oddball

Figure S8. Bar plots with 95% confidence intervals of mean accuracies in leave-one-subject-out (LOOS) cross-validation setting of MCI versus healthy aging cognition subjects using logistic regression (LR), a linear discriminant analysis (LDA), linear support vector machine (linearSVM), random forest (RFC), and deep fully-connected neural network (DFNN) classifiers. AUC, f1-scores, precision, recall, and Wilcoxon rank-sums test for significance p-values (all non-normal distributions) of the accuracy distributions above training set chance levels, which we listed above the bar plots, further supported good results of the proposed methodology. The over-sampling data augmentation (Lemaître et al., 2017) resulted in similar mean accuracies and remaining classification result metrics as in the original datasets depicted in Figure 3. Thus the over-sampling data augmentation did not significantly influence classification results.

REFERENCES

Lemaître, G., Nogueira, F., and Aridas, C. K. (2017). Imbalanced-learn: A python toolbox to tackle the curse of imbalanced datasets in machine learning. *Journal of Machine Learning Research* 18, 1–5
